# Efficacy of IANB and Gow-Gates Techniques in Mandibular Molars with Symptomatic Irreversible Pulpitis: A Prospective Randomized Double Blind Clinical Study

**DOI:** 10.22037/iej.v13i2.18625

**Published:** 2018

**Authors:** Jamileh Ghoddusi, Mohammad Hasan Zarrabi, Farzaneh Daneshvar, Neda Naghavi

**Affiliations:** a *Dental Research Center, Mashhad University of Medical Sciences, Mashhad, Iran *; b * Endodontist, Private Practice, Mashhad, Iran*

**Keywords:** Buccal Infiltration, Gow Gates Technique, Inferior Alveolar Nerve Block, Irreversible Pulpitis, Lingual Infiltration

## Abstract

**Introduction:**

The aim of the present study was to compare the efficacy of the inferior alveolar nerve block (IANB) and Gow-Gates techniques in mandibular molars with symptomatic irreversible pulpitis.

**Methods and Materials:**

In this randomised, double-blind clinical trial, 80 patients referred to Mashhad Dental School, were randomly divided into two groups: IANB and Gow-Gates anaesthetic techniques using 2% lidocaine with 1:100000 epinephrine. After injection, if pain during caries/dentin removal and access cavity preparation was reported in each group, the patients once again were randomly allocated to receive buccal or lingual supplementary infiltration. Pain severity was evaluated using a visual analogue scale. The rates of positive aspiration and changes in heart rate were compared between the IANB and Gow-Gates. Paired and individual *t*-tests and the Mann-Whitney *U*-test were used to compare the reduction in pain severity. The level of significance was set at 0.05.

**Results:**

The success rates of anaesthesia in the Gow-Gates and IANB techniques were 50% and 42.5%, respectively with no significant difference *(P*=0.562). Supplementary infiltrations significantly reduced pain severity in all subgroups (*P*<0.05). Lingual infiltration resulted in a significantly greater reduction in pain severity in the IANB group than in the Gow-Gates group (*P*<0.05). No significant difference in heart rate or positive aspiration results was observed between groups (*P*>0.05).

**Conclusions:**

In the present study, the efficacy of the IANB and Gow-Gates techniques was comparable in mandibular molars with symptomatic irreversible pulpitis. Supplementary buccal and lingual infiltration significantly reduced pain severity.

## Introduction

The inferior alveolar nerve block (IANB) is amongst the most difficult to perform local anaesthetic techniques, which is used most commonly to induce anaesthesia in mandibular molars for endodontic treatment. Clinically, the technique has been found to induce sufficient anaesthesia in 85‒90% of restorative procedures [1]. However, failure rates of 44-80% have been reported for IANB [2], and success rates are even lower (19-56%) in patients with pulpal inflammation [3-6]. Various mechanisms have been hypothesized to explain the failure of this technique in patients with irreversible pulpitis, including: decreased local pH [6], cross-innervations and accessory innervations (with lingual, buccal and mylohyoid nerve, or cervical plexus) [7, 8], because IANB does not anesthetize other branches of mandibular nerve, including lingual, buccal, and nerve to mylohyoid. A high block technique may overcome these challenges. 

IANB is achieved by injecting an anaesthetic agent into the pterygo-mandibular space. The agent is diffused in the tissue space and reaches the inferior alveolar nerve at a point immediately preceding its entrance into the mandibular foramen [9].

In 1973, Gow-Gates introduced a technique for the anaesthetisation of all mandibular nerve branches, including the inferior alveolar, lingual, buccal and mylohyoid nerves. In this technique, the anaesthetic agent is injected lateral to the mandibular condyle and beneath the lateral pterygoid muscle insertion; it reaches the nerve at its exit from the foramen oval, before its division into branches [10, 11]. 

**Figure 1:**
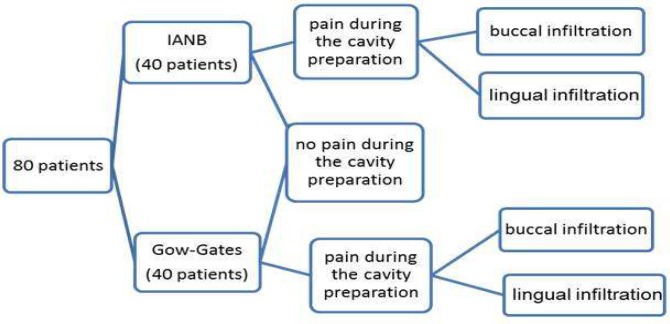
Flowchart of patient distribution

The induction of local anaesthesia is not always possible in endodontic emergencies, even with changes in technique and anaesthetic agent [2, 12, 13]. To overcome this problem, supplementary anaesthetic techniques, including intra-osseous and periodontal ligament injection [14, 15] and buccal and lingual infiltration techniques, have been recommended. These techniques may be successful alone or in combination with IANB [16-18]. The effectiveness of infiltration anaesthesia has not been tested extensively in mandibular molars. Most of the previous investigations used articaine in buccal infiltrations [19, 20]. With supporting evidence of alone infiltration of local anaesthetic solutions providing pulpal anaesthesia in up to 92% of patients with uninflamed pulp [16-18, 21], it is hypothesized that supplemental infiltration anaesthesia will affect the success rates in patients with irreversible pulpitis. Questions remain about the efficacy of lidocaine and about buccal and/or lingual position.

To our knowledge, no reported study has compared the efficacy of IANB and Gow-Gates (GG) injections, with and without buccal and lingual infiltration, in patients with irreversible pulpitis of mandibular molars. The aim of the present preliminary, prospective, randomized, double-blind study was to compare the efficacy of IANB and GG techniques with buccal or lingual infiltrations using 2% lidocaine with 1:100000 epinephrine in mandibular molars with symptomatic irreversible pulpitis.

## Materials and Methods

This randomized, parallel-grouped, double-blind clinical trial included healthy patients who presented with symptomatic irreversible pulpitis in one mandibular molar. It was approved by the Dental Research Ethics Committee of Mashhad University of Medical Sciences (MUMS) and registered at clinicaltrials.gov (identifier: NCT 01329874). All patients provided written informed consent after the nature of the procedure and possible discomforts and risks were explained fully.

Study subjects were recruited from patients referred to the Endodontic Department of MUMS. Inclusion criteria were: age of 18‒50 years, good health, provision of informed consent, moderate to severe pain in a mandibular molar, prolonged response to cold testing with cold spray (Endo-Frost; Roeko, Langenau, Germany) positive response to an electric pulp tester (EPT) (Parkell; NY, America), and vital coronal pulp on access opening. Exclusion criteria were: periapical radiolucency, active site of pathosis in the injection area, allergy to lidocaine and/or adrenalin, severe systemic disease contraindicating an endodontic procedure, pregnancy, use of medication that might affect anaesthetic assessment, inability to provide informed consent and understanding the visual analogue scale.

A power calculation dictated that a sample of 40 subjects per group would give 80% power to detect a 15% difference in the success rate of the test groups. Patients were randomly assigned to receive IANB or GG injection (40 patients each) (Figure 1).

The patients were randomly assigned to the groups by selecting a sealed opaque envelope with the group number concealed inside it. Patients in group 1 received standard IANB injections using 2% lidocaine with 1:100000 epinephrine (Persocaine; Darou Pakhsh, Iran). The first author injected the solution using 27-gauge long needles (NRK Medical Devices, Tehran, Iran). After reaching the target area, aspiration was performed and 3.6 mL (two cartridges) solution was deposited at a rate of 1 mL/min.

Patients in group 2 received GG mandibular block anaesthesia using the same anaesthetic solution. For this block, two extraoral landmarks were located: the apex of the intertragic notch and the lower border of the tragus. Each patient was asked to open his/her mouth widely, and an imaginary line was drawn from the intertragic notch to the angle of the mouth. The needle was inserted along this imaginary plane across the mesiopalatal cusp of the ipsilateral maxillary second molar. The divergence of the syringe was kept parallel to the divergence of the tragus. The same clinician injected the solution into the target area: the region lateral to the condyle neck, just below the insertion of the lateral pterygoid muscle. After bony contact, the needle was withdrawn slightly, aspiration was performed, and 3.6 mL (two cartridges) anaesthetic solution was delivered. 

Patients were asked to report when their lips were numb. An operator blinded to injection type then tested the teeth with an EPT. The teeth were tested again 15 min after injection. The canine teeth usually served as the control tooth to assess the success rate of INA block injection; if the canine did not meet the requirements for a control tooth, another tooth in the same quadrant was chosen. All data were recorded into a Microsoft Excel sheet (Microsoft Office Excel 2003). The second operator then initiated the preparation of an access opening in the involved tooth.

Supplementary local anaesthesia was administered by buccal (subgroup A) or lingual (subgroup B) infiltration of the 1.8 mL (one cartridges) of same anaesthetic solution for patients who felt pain during cavity preparation (Figure 1). In subgroup A, the solution was injected slowly (over 30 sec) buccal to the tooth apex using standard aspirating dental cartridge syringes. In subgroup B, sub-periosteal infiltration was performed at the mucogingival junction in the lingual furcation area. When complete anaesthesia and analgesia were achieved, access cavity preparation was completed and pulpotomy or pulpectomy was performed, followed by dressing with calcium hydroxide and Coltosol (Coltene Whaledent, Henan, China) as a temporary filling. When complete anaesthesia was not achieved, another type of supplementary injection was used; upon the achievement of complete analgesia, the procedures were completed as described previously, but these cases were excluded from the study.

***Determination of pain severity***

A visual analogue scale (VAS) [22] (ranging from 1 to 9) was used to determine pain severity before the procedure and during access cavity preparation after the induction of anaesthesia. The VAS was divided into three equal parts: Pain that can be tolerated (VAS: 1-3), Moderate pain that cannot be tolerated (VAS: 4-6), Severe pain that cannot be tolerated (VAS: 7-9). Each patient’s pain severity was determined using these codes. 

**Table 1:** Comparison of answer to EPT after 15 min

**Group**	**GG N (%)**	**IANB N(%)**
**Positive answer to EPT after 15 min **	8 (20)	11 (27.5)
**Negative answer to EPT after 15 min**	32 (80)	29 (72.5)
**Sum**	40 (100)	40 (100)
***P*** **-value**	*P*=0.431	

**Table 2:** Comparison of success rate between two groups

**Group**	**IANB**		**GG**	
	**Before injection** ** N (%)**	**After injection N (%)**	**Before injection** ** N (%)**	**After injection** ** N (%)**
**Complete anaesthesia /tolerable pain**	20 (50)	0 (0)	17 (42.5)	0 (0)
**Intolerable Moderate to severe pain**	20 (50)	40 (100)	23 (57.5)	40 (100)
**Sum**	40 (100)	40 (100)	40 (100)	40 (100)
***P*** **-value**	*P*=0.562			

**Table 3:** Comparison of self-reported means of pain severity before and after IANB and Gow-Gates techniques and the results of the test

**Primary injection (N)**	***P*** **-Value**	**Mean (SD) of VAS after injection **	**Mean (SD) of VAS before injection **
**Gow-Gates (40)**	*P*<0.05	2.9 (0.14)	7.07 (0.82)
**IANB (40)**	*P*<0.05	3.77 (3.59)	7.5 (0.81)

***Aspiration***

Aspiration was performed during primary injections conducted using the two tested techniques. If aspiration was positive, the case was recorded for subsequent comparison.

***Heart rate***

The local anaesthesia techniques are important factors regarding cardiovascular effects [23]. Thus, the heart rates of all patients were recorded every 30 sec from 2 min before primary injections to 5 min after secondary injections using a pulse oximeter (Oxyleth; Novametrix Co., USA). 

***Statistical analysis***

Data were recorded in a Microsoft Excel spreadsheet (Microsoft Office Excel 2003) and analysed using SPSS software (SPSS 17.0; SPSS Inc., Chicago, IL, USA). Initial and post-injection VAS scores were summarised using means and standard deviations. Paired *t*-tests were then used to evaluate the reduction in pain severity provided by each primary and supplementary technique. The Mann-Whitney *U*-test was used to compare the reduction in pain severity between the GG and IANB groups, and the *t*-test was used to compare the reductions achieved by buccal and lingual supplementary infiltration between GG and IANB groups. The *t*-test was also used to compare patients’ heart rates in different phases including pre-injection and immediately before, during, and after primary and secondary injections between the GG and IANB groups. Fisher’s exact test was used to compare aspiration results between groups. The significance level was set at 0.05.

## Results

Eighty patients (32 men, 48 women) with a mean age of 32.4 ± 8.92 years ranging from 18 to 50 years were included in the present study. Lip anaesthesia was observed a short time after injection in all cases, but large percentages of teeth still responded to an EPT (GG, 92.5%; IANB, 80%), with no significant difference between groups. Anaesthesia depth increased after the recommended waiting period of 15 min (GG, 80%; IANB, 72.5%), with no significant difference between groups (Table 1). 

**Table 4:** Comparison of the decrease in pain severity with lingual and buccal infiltration techniques between Gow-Gates and IANB groups

**Supplementary injection**	***P*** **-value**	**Mean (SD)**	**N**
**GG/ Lingual infiltration**	3.1 (1.59)	10	0.018
**IANB/ Lingual infiltration**	4.58 (1.08)	12	
**GG/ buccal infiltration**	2.74 (1.9)	11	0.617
**IANB/ buccal infiltration**	4.58 (0.9)	12	

***Pain severity***

In IANB group, success rate was 42.5%, because 42.5% of patients had no pain during caries removal and access cavity preparation (Table 2). In GG group, success rate was 50%, because 50% of patients had no pain during caries removal and access cavity preparation (Table 2). Self-reported pain severity (VAS scores) decreased significantly (*P<0.05*) after the IANB and GG techniques (Table 3), with no difference in the degree of change between groups (Table 3). VAS scores also decreased significantly after buccal and lingual infiltration following both primary anaesthesia techniques (all *P*<0.05). Supplementary lingual infiltration resulted in a significantly greater reduction in pain severity after the IANB than after the GG procedure (*P*=0.018) (Table 4), whereas the amount of reduction achieved by buccal infiltration did not differ significantly between the GG and IANB groups. 

***Heart rate and Aspiration***

No significant difference in heart rate was observed between groups. Patients’ heart rates increased significantly before injection (*P<*0.05) and returned to normal within 2 min after injection. No significant difference in aspiration findings was noted between groups (Table 5). 

## Discussion

Endodontists face the challenge of successful pain management and control. IANB is the most commonly used technique for local anaesthesia of mandibular molars. Considering the high failure rate of this technique, especially in mandibular molars with acute irreversible pulpitis, the present study was undertaken to compare the efficacy of IANB with that of the GG technique. 

The anaesthetic agent used in the present study (2% lidocaine with 1:100000 epinephrine) is the most commonly used anaesthetic agent worldwide [13]. Similar to previous studies, two cartridges of the anaesthetic agent were administered to ensure successful local anaesthesia with both techniques [24-28]. In an evaluation of the effect of local anaesthetic agent volume, Aggarwal *et al.* [29] found that the administration of 1.8 mL lidocaine with the IANB technique successfully achieved anaesthesia in 26% of cases, and the delivery of 3.6 mL anaesthetic agent was successful in 54% of cases. 

**Table 5:** Comparison of aspiration between Gow-Gates and IANB groups

**Group**	**IANB **		**GG**	
	**Number**	**%**	**Number**	**%**
**Positive aspiration**	3	3.8	2	2.5
**Negative aspiration**	77	96.2	78	97.5
**Sum**	80	100	80	100
***P*** **-value**	*P*=1			

In previous studies, two techniques have been used to evaluate the effects of local anaesthesia: pulp vitality tests, including EPT and cold tests [18, 30], and VASs [12, 31]. In the present study, a VAS was used to assess pain severity before and after injection because it has been demonstrated that lack of response to pulp vitality tests after anaesthetic agent injection does not guarantee complete anaesthesia of the pulp in teeth with irreversible pulpitis [28]. Consistent with these findings, the results of the present study showed that negative EPT results after IANB and GG injections were not completely valid [15, 32]. Fifteen min after GG and IANB injections, 80% and 72.5% of patients, respectively, had negative EPT responses. However, VAS scores indicated that only 47.5% and 40% of patients, in GG and IANB groups respectively, had achieved complete analgesia during access cavity preparation.

The success rate of anaesthesia was greater for the GG technique than for the IANB technique (47.5% *vs*. 40%), although this difference was not significant. This finding is consistent with the results reported by Goldberg *et al. *[11], but Aggarwal *et al.* [33] reported a significantly higher success rate for the GG technique than for the IANB. This difference in results might be attributed to the use of two cartridges in the study by Goldberg *et al.* [11] and present studies.

Jung *et al.* [18] showed that buccal infiltration of 4% articaine with a 1:100000 concentration of epinephrine resulted in a success rate comparable to that of the standard IANB technique in mandibular first molars with healthy pulp. Matthews *et al.* [5] used buccal infiltration of 4% articaine to supplement standard IANB in patients with inflamed pulp and achieved a success rate of 58%. Another study showed that articaine and lidocaine have comparable effects in patients with irreversible pulpitis in the posterior mandibular teeth [6]. In the present study, buccal or lingual infiltration of lidocaine was used as a supplementary injection when pain persisted after the primary injection; these supplementary techniques consistently reduced pain severity. Lingual infiltration achieved a greater reduction in pain severity after IANB than after the GG technique, whereas the amount of pain reduction did not differ according to primary injection technique for buccal infiltration.

A study conducted at Ohio University in 2000 documented an increased heart rate after the injection of etidocaine and epinephrine, which returned to normal within 4 min [34]. Similarly, we observed significant and comparable increases in heart rates in both groups, which returned to normal within 2 min. However, patients’ heart rates increased immediately before injection, likely due to anxiety and fear. This finding highlights the importance of stress-reducing protocols. However, due to the transient nature of such changes, they can be ignored in patients with no cardiac problem. 

Positive aspiration rates were 2.5% and 3.8% in the GG and IANB groups, respectively, in the present study. Watson reported positive aspiration rates of 1.6% and 3.6-22% in association with the GG and IANB techniques, respectively [35].

In conclusion, the efficacy of 3.6 mL lidocaine administered as local anaesthesia according to the IANB and GG techniques did not differ significantly in mandibular molars with acute irreversible pulpitis. Supplementary anaesthesia through buccal and lingual infiltration significantly reduced pain severity. Future studies should evaluate the efficacy of 1.8 mL lidocaine administration according to the IANB and GG techniques.

## Conclusion

In the present study, the efficacy of the IANB and Gow-Gates techniques was comparable in mandibular molars with acute irreversible pulpitis. Supplementary buccal and lingual infiltration significantly reduced pain severity. 
